# C20orf204, a hepatocellular carcinoma-specific protein interacts with nucleolin and promotes cell proliferation

**DOI:** 10.1038/s41389-021-00320-3

**Published:** 2021-03-17

**Authors:** Sebastian Burbano De Lara, Doan Duy Hai Tran, Aldrige Bernardus Allister, Mareike Polenkowski, Björn Nashan, Martina Koch, Teruko Tamura

**Affiliations:** 1 Institut fuer Zellbiochemie, OE4310, Medizinische Hochschule Hannover, Carl-Neuberg-Str. 1, D-30623 Hannover, Germany; 2 Department of Hepatobiliary and Transplant Surgery, University Medical Center Eppendorf, Martinistrasse 52, 20256 Hamburg, Germany; 3 Clinic of Hepato-Pancreatico-Biliary Surgery and the Transplantation Center First Affiliated Hospital, School of Life Sciences and Medical Center University of Sciences & Technology of China, Hefei, Anhui China; 4 Klinik für Allgemein-, Viszeral- und Transplantationschirurgie Universitätsmedizin der Johannes Gutenberg-Universität Mainz, Mainz, Germany

**Keywords:** Molecular biology, Cancer

## Abstract

In most human cancers, a large number of proteins with driver mutations are involved in tumor development, implying that multiple fine tuners are involved in cancer formation and/or maintenance. A useful strategy for cancer therapy may therefore be to target multiple cancer type-specific fine tuners. Furthermore, genome-wide association studies of tumor samples have identified a large number of long noncoding (lnc)RNA associated with various types of tumor. In this context we have previously found that C20orf204 (a splice variant of Linc00176) RNA contains a 189 amino acid (AA) long open reading frame (C20orf204-189AA) that is expressed predominantly in hepatocellular carcinoma (HCC). We report here that a protein, C20orf204-189AA, was detected in the nucleus of 14 out of 20 primary HCC, but not in control livers. Strikingly, overexpression of C20orf204-189AA enhanced cell proliferation and ribosomal RNA transcription. C20orf204-189AA is co-localized, and interacted with nucleolin via the C-terminal and with ribosomal RNA via the N-terminal domain. Furthermore, the expression of C20orf204-189AA upregulates the protein level of nucleolin. Nucleolin and C20orf204 mRNA levels in HCC are correlated with tumor differentiation grade and patient survival, suggesting that C20orf204-189AA is a cancer type-specific fine tuner in some HCC that presents itself for potential targeting therapy and cancer biomarker. Thus, cancer cells exhibit remarkable transcriptome alterations partly by adopting cancer-specific splicing isoforms of noncoding RNAs and may participate in tumor development.

## Introduction

A large number of driver mutations have been reported in most human cancers. For example, it has been recently shown by exome sequencing of hepatocellular carcinoma (HCC) that 161 putative driver genes are associated with 11 recurrently altered pathways in HCC development^1^, suggesting that many signaling pathways are altered to a modest degree, and act together. Here, genome-wide association studies of tumor samples have identified a large number of long noncoding (lnc)RNAs associated with various types of cancer^2^. These facts suggest that multiple target molecules including noncoding RNA will be required for effective cancer therapy. Furthermore, recent ribo-sequencing data suggest that 40% of lncRNAs and pseudogenes are potentially translated into peptides with an average size of 43 amino acids in human cells^3^. These peptides may be useful biomarkers for cancer diagnosis. Furthermore, these micropeptides derived from lncRNAs may have a biological function^4^.

We have recently shown that long intergenic noncoding (*Linc)00176/C20orf204* is activated by the proto-oncogene transcription factor Myc and is expressed at high levels in HCC^5^. *Linc00176/C20orf204* is expressed at a low level or is not transcribed in normal human liver or in other types of human organs and cells, such as pancreas, heart, B cell, skin, lung, temporal brain lobe, muscle, mesenchymal Wharton’s Jelly, mesenchymal adipose, mesenchymal bone marrow, H7-hESC, or in other cancer cell lines, such as K562, A375, MCF-7, SK-N-DZ, SJCRH30, or HeLa cells^5^. Importantly, the expression level of *Linc00176/C20orf204* correlated both with the differentiation grade in primary HCCs and with the survival time of HCC patients (the cancer genome atlas (TCGA) data (https://cancergenome.nih.gov/))^5^. *Linc00176/C20orf204* was originally identified from the human oligodendroglioma cDNA library, NCI_CGAP_Brn67 (IMAGE ID: 4941074). We have recently shown that 1590 nucleotides in a middle part of *Linc00176* Exon 2 are spliced out in HCC, resulting in the formation of an 189 amino acid long open reading frame^5^. In the NCBI and Ensembl (ENST) data base, five variants of *Linc00176/C20orf204* namely NM_001348090.1, XM_024451876.1, ENST00000444463.5, ENST00000431158.1 and ENST00000636176.1 are listed, although ENST00000444463.5 and EENST00000431158.1 contain the whole Exon 2, and as a result just a 79 amino acid long open reading frame at a different part of Exon 2 than in the HCC-specific splice variant of Linc00176 (Fig. S1). *Linc00176* was re-named as *C20orf204* (the gene name is designated by the chromosome of origin, with the letters “orf” for open reading frame and a number in a series) by the HUGO gene Nomenclature Committee. Whether the gene product is indeed translated was not known.

In this report, we show that C20orf204 is translated in HCC (C20orf204-189AA). C20orf204-189AA enhanced HCC proliferation and ribosomal RNA transcription and interacts with nucleolin and ribosomal RNA, indicating that this molecule is one of the cancer-specific fine tuners for HCC formation.

## Results

### *C20orf204* in HCC cell lines is translated into a 189 amino acid long arginine rich protein

**Fig. 1:**
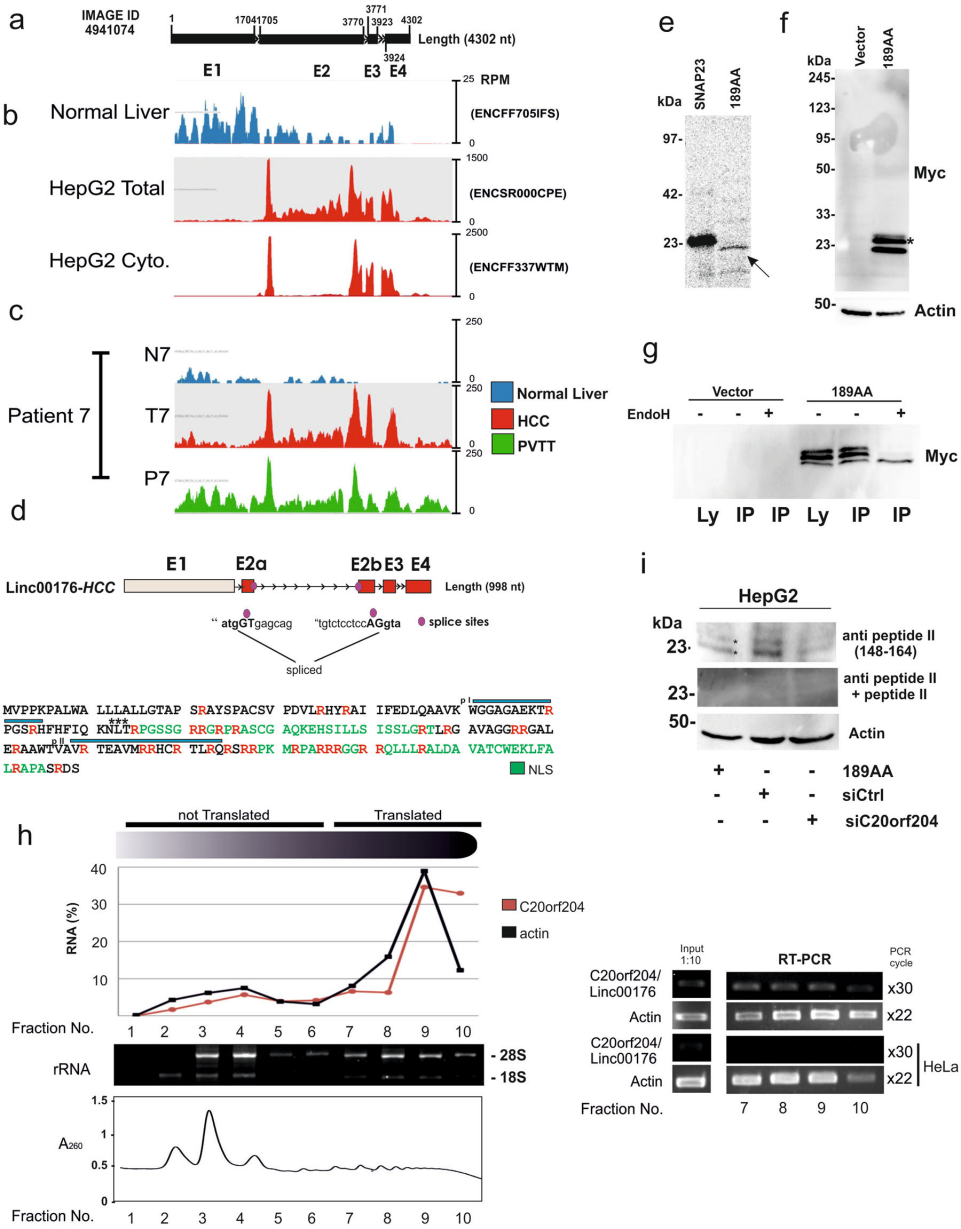
*C20orf204/Linc00176* transcript in HCC lacks Exon 1 and a part of Exon 2, and as a result contains 189 amino acid long open reading frame. **a** Schema of *C20orf204/Linc00176* (IMAGE ID 4941074). **b** The HCC-specific splice variant of *Linc00176, C20orf204* contains an 189 amino acid long potential open reading frame (C20orf204-189AA). Total RNA-seq datasets from human liver tissue (ENCFF705IFS), HepG2 cells (Total: ENCSR000CPE; cytoplasmic: ENCFF337WTM), primary HCC (T) (patient 7), PVTT (P), and corresponding normal liver (N) (GSE77509) were aligned to the reference human genome (Hg38). SeqMonk was used to quantitate and visualize the data. Blue, red and green peaks in the wiggle plot represent the normalized RNA-seq read coverage on *C20orf204/Linc00176*. RPM: reads per million. **c** Predicted *C20orf204/Linc00176* that is expressed in HepG2 cells (*C20orf204-HCC*). Red boxes: transcribed at high level; gray boxes: transcribed at low level in HCC. Numbers represent nucleotide number. Closed purple circles represent splice sites (1894 and 3495) in Exon 2 (E2a: 1705–1894; E2b: 3495–3770). Protein sequence-derived HCC-specific splice variant (E2a–E2b–E3–E4) from HepG2 and Huh7 cells. p-I, p-II (blue lines): positions of peptide I and peptide II that were used to generate rabbit antibodies. ***: A potential *N*-glycosylation site. **e** In vitro transcription/translation assay of C20orf204, proteins are labeled with [^35^S]methionine. Synaptosomal-associated protein 23 (SNAP23), a vesicular transport protein was used as a positive control^25^. **f** Myc-tagged C20orf204-189AA (189AA) and empty vector pcDNA3MycHis (Ctrl) were transfected in HeLa cells, and Myc-specific immunoblot was performed. **g** Parallel culture of **f** was immunoprecipitated with Myc antibody (IP) and then immunoprecipitates were incubated with EndoH. **h** HepG2 cytoplasmic lysate was prepared and fractionated on sucrose gradients. The distribution of RNA was calculated using the CT values obtained by qRT-PCR. Isolated RNA was supplied in a gel to determine translated fractions. mRNAs were prepared from the indicated fractions and were applied for actin or C20orf204 specific RT-PCR. X: PCR cycle. **i** HepG2 cells were transfected siCtrl and siC20orf204 and then supplied for immunoblot using antipeptide (148–164) antibody with or without peptides. As a positive control Myc-tagged C20orf204-189AA (Myc-189AA) was expressed in HeLa cells. Three independent experiments were performed.

We have previously shown that a splice variant of *Linc00176* (IMAGE ID: 4941074), *C20orf204* in HCC cell lines, HepG2 and Huh7, is transcribed into a 998 nucleotide (nt) long transcript and obtains an open reading frame of 189AA via splicing of the middle part of Exon 2 (Fig. 1a, b)^6^. To examine whether other transcript variants of *Linc00176* are expressed, we analyzed Cap analysis gene expression sequencing (CAGE-seq) generated by ENCODE Consortium. CAGE-Seq detects a strong signal near to the transcription start site of the *C20orf204* variant but not for other variants (Fig. S1), suggesting only the C20orf204 variant is expressed in HepG2 cells. *C20orf204* has an FPKM of 87.81 in HepG2 cells and can also be detected in primary HCCs. RNA-seq data from primary HCC, portal vein tumor thrombosis HCC and corresponding normal liver (GSE77509)^7^ revealed that *C20orf204* is detected in some primary HCCs, but not in corresponding normal liver (Fig. 1c). Notably, this protein, C20orf204-189AA, contains multiple nuclear localization sequences, potential *N*-glycosylation site (72–74, amino acid number) and contains 32 arginine residues (Fig. 1d). Next, we performed an in vitro transcription/translation assay with C20orf204-189AA, revealing a single band at 22 kDa (Fig. 1e, arrow). Furthermore, to examine whether C20orf204 protein is stable in cells, we transfected HeLa cells with myc-tagged C20orf204-189AA and performed a Myc-specific immunoblot (Fig. 1f). In addition to a 22 kDa band, bands with a molecular mass of 26 and 24 kDa were detected in C20orf204-189AA-Myc-expressing cells (Fig. 1f). Since C20orf204-189AA contains a potential *N*-glycosylation site, C20orf204-189AA-expressing cell extracts were immunoprecipitated by Myc antibody and were then incubated with glycosidase EndoH. After incubation the upper two bands were no longer detected, indicating that C20orf204-189AA may be partially glycosylated (Fig. 1g). These data indicate that C20orf204-189AA is stable in cells. To examine whether C20orf204-189AA is endogenously translated in HepG2 cells, we isolated the polysome fraction of HepG2 cells using sucrose gradient centrifugation^8^. The *C20orf204* transcript is mainly detected in translated fractions of HepG2 cells (Fig. 1h), strongly suggesting that a part of C20orf204-189AA is endogenously translated.

These data suggest that C20orf204 protein is translated in an HCC-specific manner, implying that C20orf204-189AA could be a potentially suitable cancer biomarker and/or participate in tumor development. To examine these points, we next generated a rabbit antibody against two mixed synthetic peptides corresponding to amino acid positions 51–65 (peptide I) and 148–164 (peptide II) of C20orf204-189AA (Kaneka Eurogentec S.A. Belgium) (positions are shown in Fig. 1d), and then examined endogenous C20orf204 expression in HepG2 cells.

By immunoblot using antipeptide I and peptide II antibodies, proteins with molecular masses of 22, 24 and 26 kDa were detected. These bands were not detected by peptide absorbed antibody or after depletion of C20orf204 (Fig. 1i).

### The protein, C20orf204-189AA, is present in some primary HCCs

**Fig. 2:**
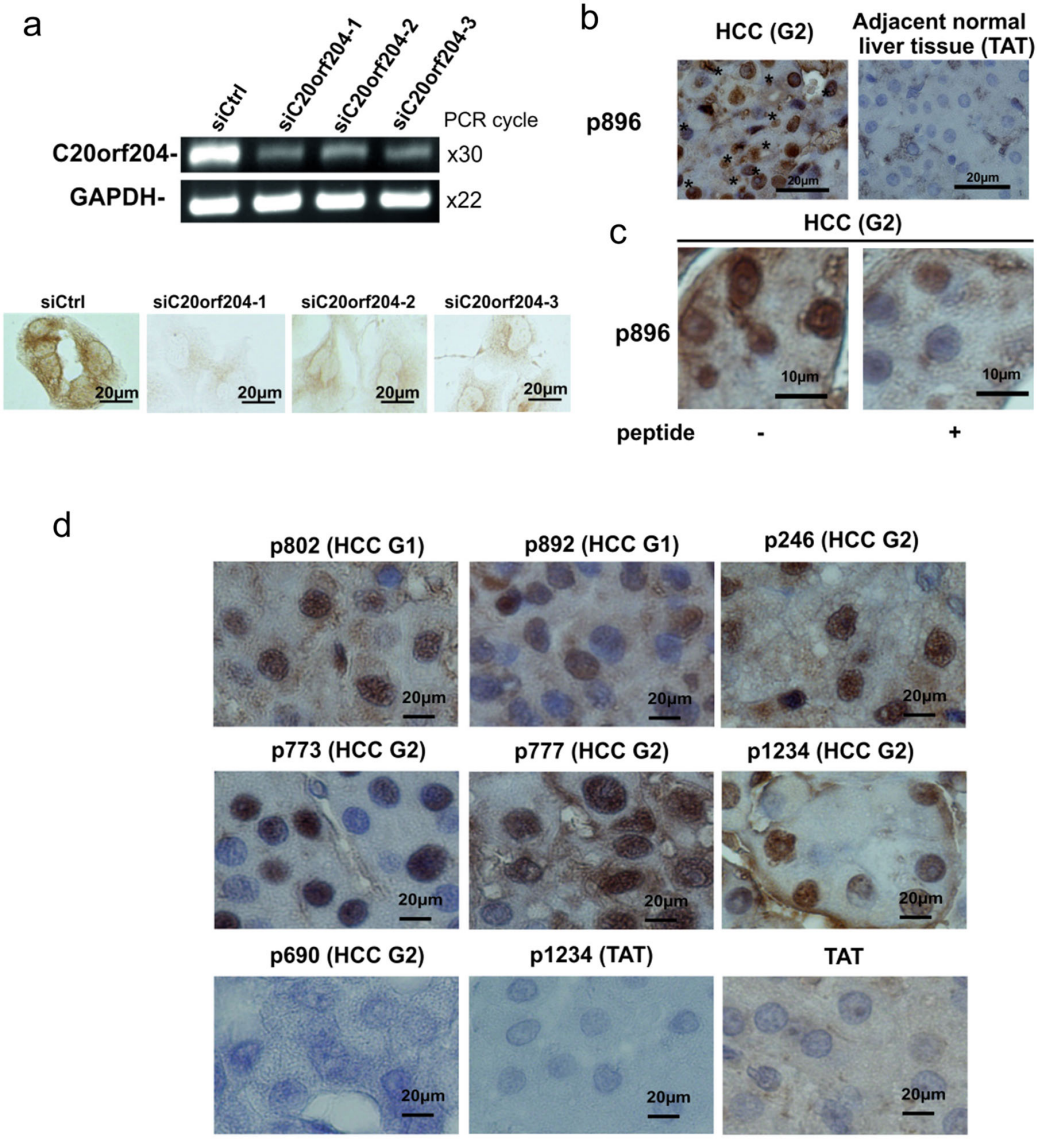
C20orf204-189AA was detected in HepG2 cells and human primary HCCs. **a** HepG2 cells were transfected with siCtrl, siC20orf204-1, siC20orf204-3, and siC20orf204-3, and RNAs were supplied for *C20orf204*, or *GAPDH-*specific RT-PCR or were fixed and then stained by C20orf204-peptide-specific IHC. **b** Paraffin sections from p896 HCC and its control liver samples were stained with rabbit antibody against C20orf204-peptide I and peptide II and hematoxylin. *Nuclear localization of C20orf204-product. C20orf204 protein is located in nucleus; however, the corresponding normal liver sample (TAT) was not stained. **c** Intensity of C20orf204 protein staining was drastically reduced using antibody pre-absorbed with peptides. **d** Additional C20orf204 positive and negative primary HCCs and TAT were stained by peptide-specific antibody. The representative images of each category are shown. All bars represent 20 µm.

To examine whether C20orf204-189AA-specific peptide antibody can be used for immunohistochemically (IHC) staining, HepG2 cells were stained with the peptide-specific antibodies. In HepG2 cells, C20orf204-189AA protein was detected in the nucleus and in the perinuclear regions (Fig. 2a). Upon depletion of *C20orf204* using three different siRNA, the staining intensity was drastically reduced (Fig. 2a). We then examined HCC samples and tumor adjacent normal liver tissues (TAT) from Indivumed GmbH (Hamburg, Germany). The nucleus of primary HCC from p896 is clearly stained with antibody against the mixture of peptide I and II, while no staining was observed in corresponding normal liver tissue (Fig. 2b). The nuclear staining in p896 HCC was drastically reduced using antibody that was pre-absorbed by peptides (Fig. 2c), indicating that nuclear staining is peptide antibody specific. We examined 20 primary HCC and 7 TAT (Table 1). Nuclear staining was observed in 14 samples of HCC, but not in any of the TAT samples. Interestingly, cytoplasmic staining was seen in two of the seven TAT samples. The role of C20orf204-189AA in the cytoplasm is unknown. Representative data for C20orf204-189AA positive and negative results in primary HCC and TAT are shown in Fig. 2d. These data suggest that C20orf204-189AA may be a potential HCC biomarker; however, more samples will be required for this investigation.

**Table 1:** Patient samples supplied for C20orf204-189AA immunohistochemistry using antibody against peptide I and peptide II mix.

Samples		Nuclear staining				Cytosolic staining			
Tissue type	Differentiation grade	Positive	Total	%	*P* val.	Positive	Total	%	*P* val.
Tumor	G1	5 (6)	14 (20)	70	736E−02	3 (6)	9 (20)	45	655E−01
	G2	8 (12)				4 (12)			
	G3	1 (2)				2 (2)			
Non-tumor	TAT	0 (5)	0 (7)	0	815E−03	1 (5)	2 (7)	28.57	257E−01
	TAT^a^	0 (2)				1 (2)			

^a^TAT from adenoma carrying patients.

### C20orf204-189AA enhanced cell proliferation

**Fig. 3:**
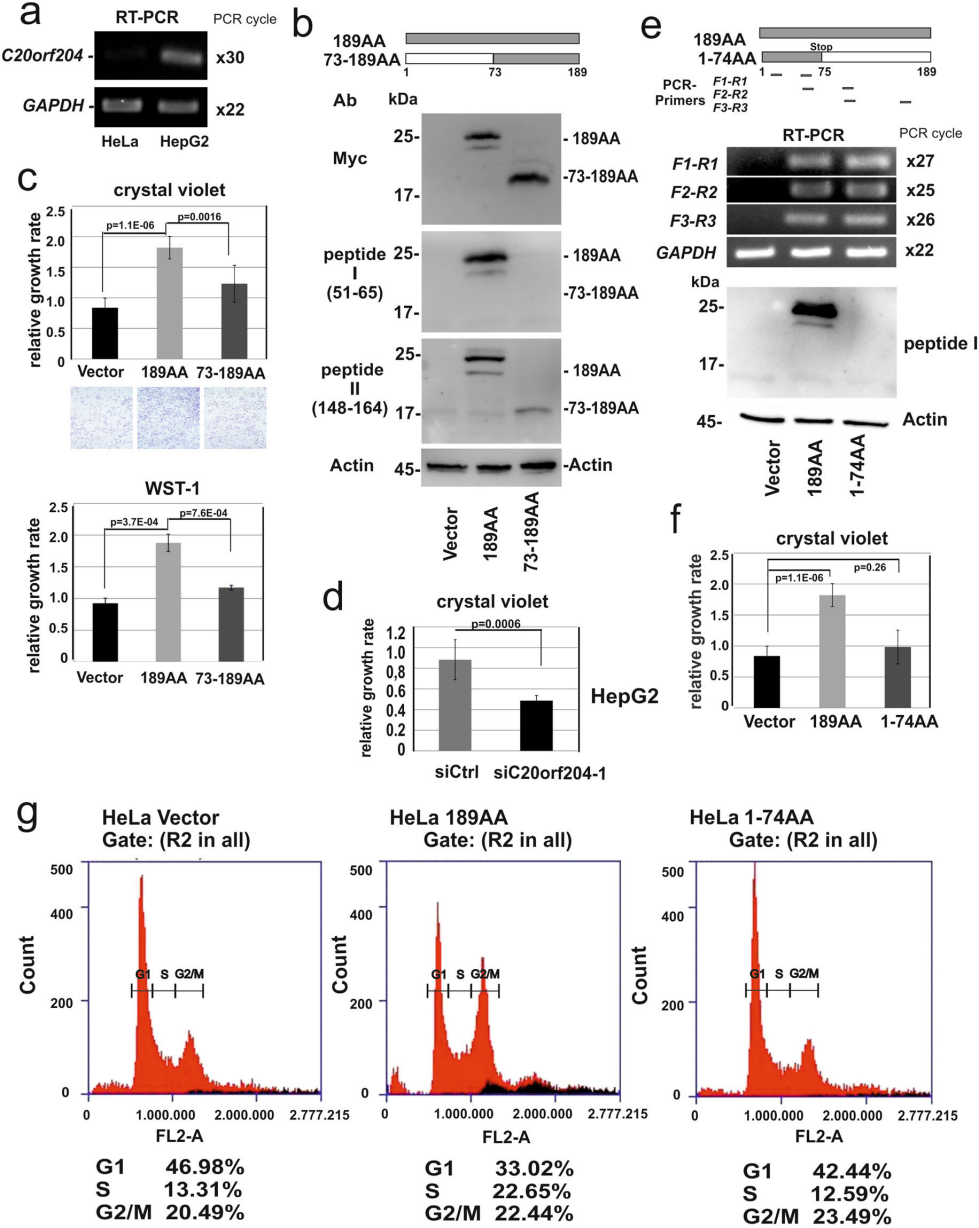
Ectopic expression of C20orf204-189AA enhances cell proliferation. **a** RNA from HeLa or HepG2 cells were applied for C20orf204 and GAPDH-specific RT-PCR. **b**, **c** HeLa cells were transfected with pcDNA3MycHis alone (Vector), or vector carrying with C20orf204-189AA or C20orf204-(73-189AA). Then, Myc-, peptide I (51–65)-, peptide II (148–164)- or actin-specific immunoblot was performed (**b**) and the parallel cultures from **b** were supplied for crystal violet staining or WST-1-assay (**c**). **d** HepG2 cells were transfected with si*Ctrl* and si*C20orf204* and then were supplied for crystal violet staining. **e** HeLa cells were transfected with *C20orf204-189AA* or the mutant carrying stop codon “TGA” at position 75AA “AGA” (C20orf204-(1-74AA)) and RNAs were supplied for *C20orf204*-specific RT-PCR using three different position (Table 1) primer pairs. A parallel culture was supplied for immunoblot against peptide I. **f**, **g** The parallel cultures of **e** were supplied for crystal violet staining (**f**) or PI staining followed by FACS analysis (**g**) 3 days after transfection as described in “Materials and methods”. Three independent experiments were performed.

We next examined whether C20orf204-189AA associates with biological function(s). Since HeLa cells do not express C20orf204-189AA (Fig. 3a), Myc-tagged C20orf204-189AA in HeLa cells was expressed (Fig. 3b). We next examined whether C20orf204 influences cell growth by the crystal violet staining assay and WST assay (Fig. 3c). To analyze a potential functional domain, we include an N-terminal deletion mutant C20orf204-(73-189AA) that is also stable in the cells (Fig. 3b) in this experiment.

Here, within 3 days, growth of C20orf204-189AA-overexpressing HeLa cells was approximately twofold (*p* = 1.1E−06 by crystal violet assay, and *p* = 3.7E−04 by WST assay) greater than control vector transfected HeLa cells (Fig. 3c); however, deletion of the first 72 amino acids of C20orf204-189AA clearly reduced the enhancement of cell growth (*p* = 0.0016 and *p* = 7.6E−04). Furthermore, depletion of *C20orf204* RNA in HepG2 cells reduced cell proliferation approximately twofold within 3 days (Fig. 3d), suggesting again that C20orf204 promotes cell proliferation. In addition, we overexpressed C20orf204-189AA in Huh7 cells, a differentiation grade G1 HCC cell line that expresses C20orf204 at low level^5^, and these cells also showed an increase in proliferation (1.5-fold; *p* = 0.002) (Fig. S2). Previously, we have shown that *C20orf204/Linc00176* RNA has a sponge function for tumor suppressor microRNAs, miR-9-5p and miR-185-5p. Using the program MIRANDA 3.3a (parameters: score 140, energy −7, scale 7)^9^ by screening with 544 high confidence microRNAs (https://www.mirbase.org) C20orf204-HCC (998 nt long) is shown to contain 74 and 84 potential binding sites for miR-9-5p and miR-185-5p, respectively. Upon depletion of *Linc00176/C20orf204* in HepG2 or Huh7 cells, both cells underwent cell death; however, inhibitors of miR9 and miR189 rescued cells from cell death^5^. Notably, *C20orf204-189AA-Myc* RNA still exhibits 14 and 12 potential binding sites for miR9 and miR189, respectively. To examine whether enhancement of proliferation is due to the 189AA protein or regulatory function of *C20orf204 RNA*^5^, we added a stop codon “TGA” after 74 AA position (*C20orf204-(1-74AA)*). Both *C20orf204-(1-74AA)* and *C20orf204-189A-Myc* exhibit 14 and 12 potential binding sites for miR9 and miR189, respectively. After transfection of HeLa cells with *C20orf204-189AA* or *C20orf204-(1-74AA)* cDNA, we performed *C20orf204*-specific reverse transcriptase PCR (RT-PCR) using three different primer pairs. Both wild type and mutant RNAs were expressed to the same extent (Fig. 3e); however, although ectopic expression of C20orf204-(1-74AA) slightly increases cell growth (1.2-fold, *p* = 0.26) (difference is not significant, but this difference may be due to the miR sponge function), expression of C20orf204-189AA was enhanced significantly (more than twofold, *p* = 1.1E−06), indicating that the protein plays a role in enhancement of cell growth not RNA sponge function (Fig. 3f).

Furthermore, cells were labeled with propidium iodide (PI) and were analyzed by FACS. Strikingly, the S peak of C20orf204-189AA overexpressing cells was 1.7-fold enhanced, while the G1 peak was reduced to 70% compared to control vector transfected cells. In agreement with the cell growth assay, C20orf204-(1-74AA) expressing cells showed a similar distribution of S and G1 peaks to that of control cells (Fig. 3g), implicating that enhanced proliferation in HeLa cells is due to the C20orf204-189AA protein, not the RNA. Notably, N-terminal deletion mutant C20orf204-(73-189AA), but not C20orf204-(1-74AA), is stable in cells (Fig. 3b, e), suggesting that the C-terminal domain is essential for protein stabilization in vivo.

### C20orf204-189AA is co-localized and interacts with nucleolin and rRNA

**Fig. 4:**
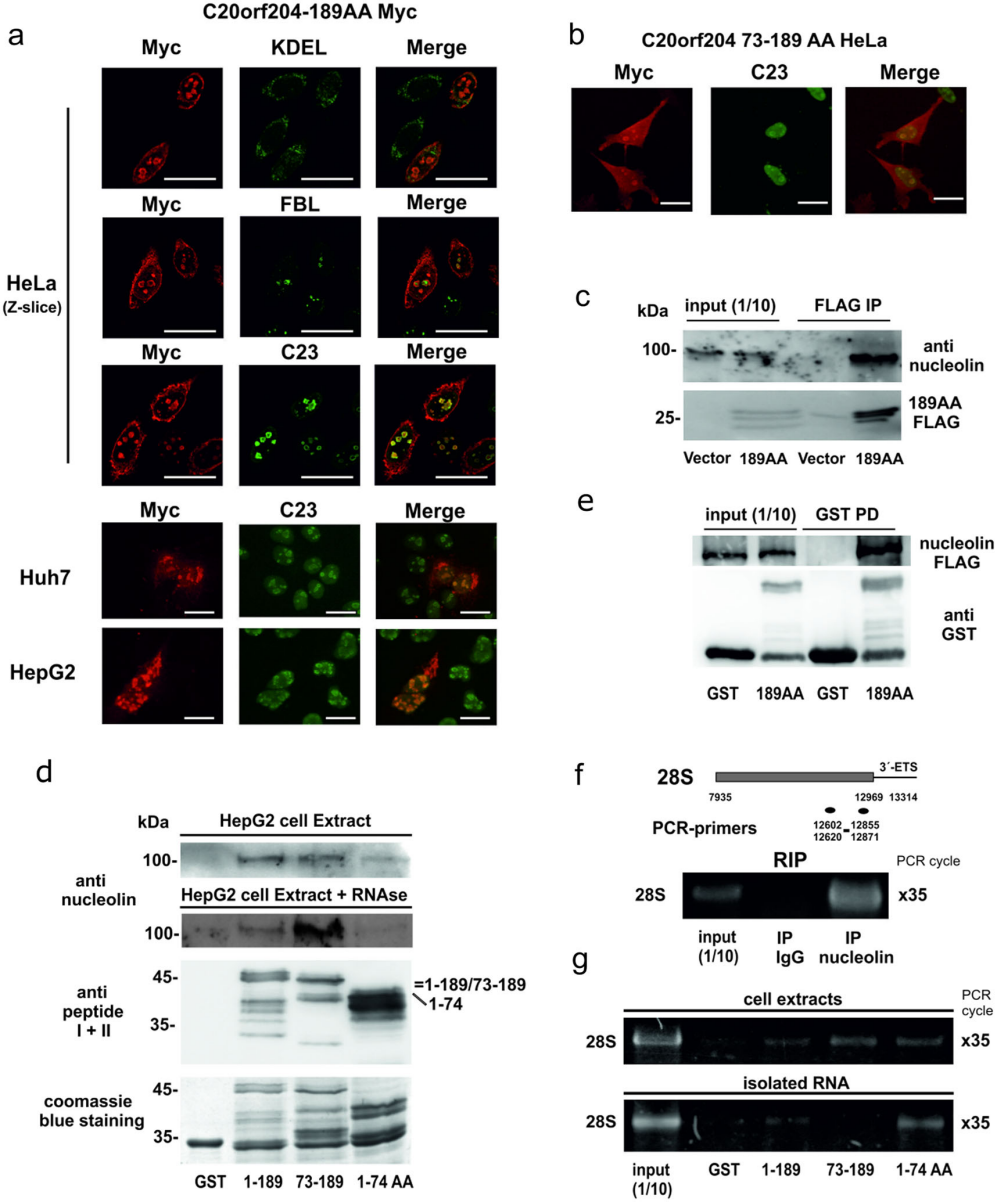
C20orf204-189AA is co-localized and interacted with nucleolin. **a**, **b** Myc-tagged C20orf204-189AA was expressed in HeLa, HepG2, and Huh7 cells (**a**) or its deletion mutant, Myc-tagged C20orf204-(73-189), was expressed in HeLa cells (**b**). Then, cells were stained with anti-Myc and nucleolin, KDEL, or FBL specific antibodies and visualized by the immunofluorescent (IF) technique. Z-slice: visualized by confocal microscopy. Three independent experiments were performed and representative images are shown. All bars represent 20 µm. **c** FLAG-tagged C20orf204-189AA (189AA) was expressed in HeLa cell; cell extracts were precipitated by FLAG antibody, and then nucleolin-specific immunoblot was performed. Vector: extracts from control vector transfectants. **d** GST, GST-C20orf204-189AA, C20orf204-189AA-(73-189) or C20orf204-(1-74) were isolated (Coomassie blue staining) and then incubated with HepG2 cell extracts, with and without RNAse, and bound proteins were analyzed by immunoblot using nucleolin, or peptide antibody. **e** FLAG-tagged nucleolin was expressed in HeLa cells, cell extracts were precipitated by FLAG antibody and incubated with isolated GST, GST-C20orf204-189AA, C20orf204-189AA-(73-189) or C20orf204-(1-74) and then FLAG and GST specific immunoblot was performed. **f** HepG2 cell extracts were precipitated with control IgG (IgG) or nucleolin antibody and then bound RNAs were analyzed by 28 rRNA-specific RT-PCR (RNA immunoprecipitation (RIP)). ETS external transcribed spacer. **g** GST and its fusion proteins described in **d** were incubated with HepG2 cell whole extracts (cell extracts) or isolated RNAs (RNA) and bound RNAs were analyzed by RT-PCR using 28S rRNA- specific primers. Three independent experiments were performed.

To examine how C20orf204-189AA regulates cell proliferation, we first examined where the C20orf204-189AA is located intracellularly. HeLa cells and the HCC cell lines, Huh7 and HepG2, were transfected with C20orf204-189AA-Myc cDNA and Myc-tagged C20orf204-189AA were stained using the immunofluorescent technique by a Myc-specific antibody. Since C20orf204-189AA was detected in nucleoli-like regions, we further co-stained with the nucleolar marker nucleolin (C23). In all three cell lines, C20orf204-189AA is co-localized with nucleolin. C20orf204-189AA is also co-localized with fibrillarin (FBL) a small nucleolar protein that participates in rRNA processing, indicating that the C20orf204-189AA is mainly in nucleoli (Fig. 4a). However, some C20orf204-189AA localizes to the ER as shown by co-staining with the ER marker KDEL (a target peptide sequence that prevents proteins from leaving the ER) antibody (Fig. 4a). The N-terminal deletion mutant (C20orf204-(73-189AA)) that did not enhance cell proliferation is localized mainly in the cytoplasm (Fig. 4b), suggesting that C20orf204-189AA in nucleoli participates in cell proliferation. Here, C20orf204-189AA is not only co-localized, but also Flag-tagged C20orf204-189AA co-precipitated with nucleolin (Fig. 4c). Furthermore, a glutathione-*S*-transferase (GST) pull-down assay revealed that nucleolin binds to GST-C20orf204-189AA, and GST-C20orf204-(73-189AA), but not to GST alone. Nucleolin also binds to C20orf204-(1-74AA) but to a much lesser extent (Fig. 4d), suggesting that (73-189AA) is the main binding site for nucleolin. In the presence of RNAse, C20orf204-(73-189AA) clearly binds to nucleolin, indicating that the C-terminal domain plays a role in the interaction of nucleolin and C20orf204-189AA (Fig. 4d). RNAse treatment abolishes the interaction of C20orf204-(1-74AA) with nucleolin, indicating that weak binding of this domain to nucleolin is via RNA. Furthermore, flag-tagged purified nucleolin binds to purified GST-C20orf204-189AA fusion protein (Fig. 4e), indicating that these two proteins directly interact with each other.

Nucleolin has been shown to be implicated in multiple steps of ribosome biogenesis such as the synthesis of rRNA by RNA pol I and rRNA processing^10^ and directly binds to rRNA. We confirmed the binding of nucleolin to rRNA in HepG2 cells. Indeed, the nucleolin antibody precipitated 28S rRNA in this system (Fig. 4f, IP nucleolin). Then, we examined whether C20orf204-189AA influences binding of nucleolin to rRNA. Whole-cell extract of HepG2 cells were incubated with GST, and with GST fusion proteins and bound RNA was then isolated. rRNA was detected with all GST fusion proteins, but not with GST alone. Since 28S rRNA was detected also in the GST-C20orf204-(1-74AA)-bound fraction, we incubated isolated RNA directly with all GST fusion proteins. Here, rRNA 28S was clearly detected in GST-C20orf204-(1-74AA) (Fig. 4g, RNA).

Taken together our results suggest that C20orf204-189AA binds to nucleolin and rRNA forming a trimer complex.

### C20orf204-189AA stabilizes nucleolin and promotes rRNA transcription

**Fig. 5:**
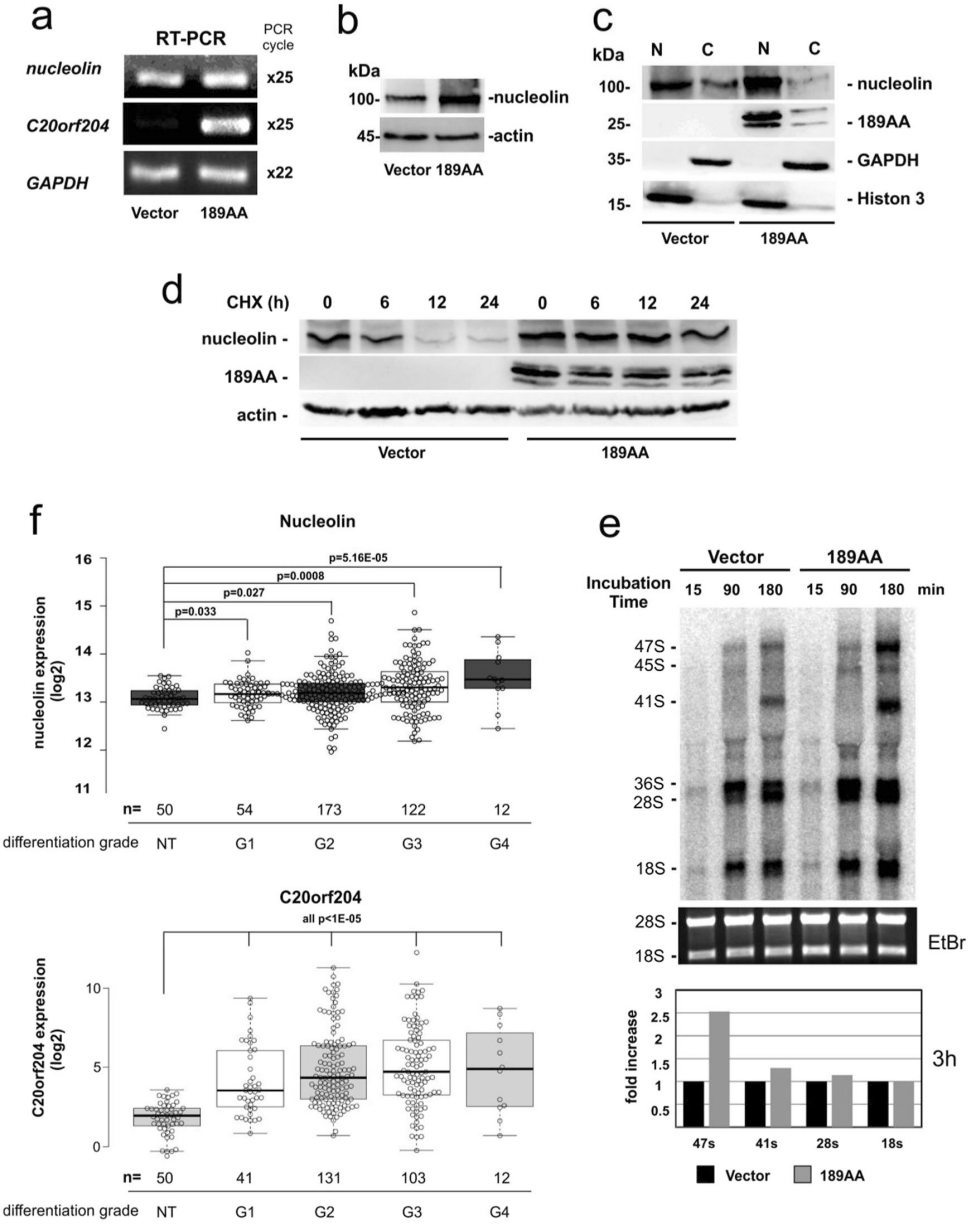
C20orf204-189AA increases the level of nucleolin and nucleolin expression level is correlated with differentiation grade and survival time in HCC patients. **a**, **b** HeLa cells transfected with control vector or vector carrying C20orf204-189AA cDNA. Cells extracts were analyzed by *nucleolin, C20orf204*, or *GAPDH* specific RT-PCR (**a**) or nucleolin and actin specific immunoblot (**b**). **c** A parallel culture of **a** was separated into nuclear (N) and cytosolic (C) fractions. The fractions were supplied for nucleolin, myc, GAPDH, and histone 3-specific immunoblots. **d** C20orf204-189AA was expressed in HeLa cells. After 24 h cyclohexamide (CHX) 10 µg/ml was given. Parallel cultures were lysed at different times and supplied for nucleolin or myc specific immunoblots. **e** HeLa cells were transfected with control vector or C20orf204-189AA. After 24 h the cells were incubated in phosphate-free DMEM for 2 h and [32P]orthophosphate was added to the medium (125 mCi/ml) for the indicated times. The ^32^P-labeled RNAs were isolated and supplied for analysis. **f** Expression of *nucleolin* and *C20orf204* in differentiation grade G1–G4 HCC (*n* = 361) samples. Data obtained from cbioportal (https://www.cbioportal.org/) shown as box blot.

To examine whether C20orf204-189AA exerts an influence on the level of nucleolin expression, we compared *nucleolin* mRNA and nucleolin protein in C20orf204-expressing HeLa cells and control cells. There is no significant difference in the mRNA level (Fig. 5a); however, the protein level in nucleus is increased when C20orf204 was expressed (Fig. 5b, c). Furthermore, cells expressing C20orf204-189AA and control cells were stained by nucleolin-specific antibody. To quantitate the intensity of nucleolin staining reciprocal pixel intensity was used. Nucleolin levels are approximately twofold enhanced (Fig. S3). Since nucleolin displays multiple subcellular localizations, we investigated the influence of C20orf204-189AA on the appearance of nucleolin in the nucleus and cytoplasm. Our data reveal that nuclear nucleolin levels are elevated when overexpressing C20orf204-189AA (Fig. 5c). Consequently, we supplied cyclohexamide (CHX)-treated nuclear cell lysates to investigate nucleolin half-life with and without C20orf204-189AA overexpression. After 16 h nucleolin protein levels are clearly reduced in control cells; however, they remain constant in C20orf204-189AA-overexpressing cells (Fig. 5d). Since nucleolar nucleolin plays a key role in rRNA synthesis, we analyzed rRNA levels in C20orf204-189AA transfected and empty vector transfected HeLa cells. Cells were labeled with the indicated times. After 3 h incubation, ^32^P-labeled RNA was analyzed by agarose gel electrophoresis. Clearly, the level of 47S is increased approximately 2.5-fold more in C20orf204-189AA-overexpressing cells than in control cells (measured by reciprocal pixel intensity). Our results show an increase in rRNA transcription when compared to non-transfected cells (Fig. 5e). These data and our previous results suggest that C20orf204-189AA stabilizes nucleolin and promotes rRNA transcription and proliferation. In addition, since nucleolin plays a role in cell immortalization^11^, C20orf204-189AA may also be involved in cancer cell immortalization. These data suggest that C20orf204-189AA participates in HCC development via nucleolin stabilization and rRNA transcription. The role of directly bound rRNA on C20orf20-189AA is unknown.

### The nucleolin expression level is correlated to differentiation grade and survival time in HCC patients

We previously showed that the Linc00176/C20orf204 expression level is correlated with the survival rate and differentiation grade in HCC patients using the TCGA data base^5^. In this context, here we present expression levels of both *Linc00176/C20orf204* and *nucleolin* in HCC. Both expression levels correlate with HCC differentiation grade in primary HCCs suggesting that both could participate in cancer maintenance (Fig. 5f). The expression level of nucleolin is also significantly correlated with HCC patient survival time. Here, we analyzed data using Kaplan–Meier estimation. Correlation between *nucleolin* expression level (log2 RSEM (RNA-Seq by Expectation-Maximization) ≥13.5 (*n* = 82): log2 RSEM < 13.5 (*n* = 289)) and survival time (within 7.5 years) is significant (Fig. S4) (log-rank test: *p* = 4.070E-2).

## Discussion

We have previously shown that whereas depletion of three fine tuners in HCC: transmembrane BAX-inhibitor motif-containing 4 (TMBIM4), transmembrane emp24-like trafficking-protein 10 (Tmed10), and D-tyrosyl-tRNA deacylase 2 (Dtd2), induces cell death in HCC cells, depletion of just one or two of these does not^12^. In this report, the novel protein C20orf204-189AA that is expressed predominantly in HCC participates in HCC cell growth and stabilizes nucleolin. It has been shown that nucleolin participates in the formation of multiple cancers, such as breast cancer or T cell lymphoma^11^. Here, in agreement with TGCA data, survival of 147 HCC patients at Kaohsiung Chang Gung memorial Hospital (Taiwan) is correlated with the *nucleolin* expression level^13,14^.

Endogenous and exogenous C20orf204-189AA is detected as a protein with a molecular mass of 26, 24, and 22 kDa. Our data suggest that the protein is partially glycosylated; however it is also localized in nucleoli. In this context, nucleolin has also been described as a shuttling molecule between the nucleus, the cytosol, and the cell surface and is partially glycosylated^8^.

*C20orf204* was originally identified as a splice variant of *Linc00176* (ref. ^5^) and both RNA and protein participate in tumor development. Here, most long noncoding RNAs are cell type specific^15^, and several alternative splicing factor genes such as SRSF10, PTBP2, or HNRNPM are upregulated in HCC^16^, suggesting that the alternative spliced lncRNAs may be detectable in HCC-specific variants. This also indicates the potential presence of the specific alternative spliced lncRNAs in HCC. Here, recent ribo-sequencing data suggest that 40% of lncRNAs and pseudogenes are potentially translated into peptides with an average size of 43 amino acids in human cells^3^. These peptides may be useful biomarkers for cancer diagnosis. Furthermore, these micropeptides derived from lncRNAs may have a biological function^4^. It has been shown that a micropeptide is involved in muscle performance^17^ and growth^18^. In addition, SPAR polypeptides encoded by the Linc00961 regulate mTORC1 and muscle regeneration^19^, and another micropeptide, mitoregulin, is involved in protein complex assembly in mitochondria^20^. Thus, additional cancer-specific fine tuners may be found stemming from cancer-type-specific lncRNA-derived peptides.

In addition, cancer cells exhibit remarkable transcriptome alterations partly by adopting cancer-specific splicing isoforms. These isoforms of lncRNAs may exhibit a long open reading frame like C20orf204 and may participate in cancer cell maintenance. Here, it has been shown that tumor-specific splice variants of coding RNA also participate in growth, development, and progression of therapy-resistant tumors^21,22^.

These finding offer novel biomarkers originated from noncoding RNAs and splice variants as well as a novel strategy for cancer treatment that targets multiple cancer type-specific fine tuners.

## Materials/subjects and methods

### Cell culture, siRNA, and transfection

HepG2, Huh7, and HeLa cells were purchased from the American Type Culture Collection (Manassas, VA) or the DMSZ-German collection of microorganisms and cell culture (Braunschweig, Germany). They were grown in DMEM supplemented with 10% FCS. All cell lines are free of mycoplasma contamination.

*Control siRNA* 1 (sc-37007) (Santa Cruz, CA, USA), *control siRNA-2* (5′-UAAGGCUAUGAAGAGAUAC-3′), *siC20orf204-1, -2*, and -*3* (5′-CUCGUUCUGUAGACUUGUU-3′, 5′-GGCCUCAAUAAACGGAGCU-3′ and 5′-AAACCUGACUAGACCCGGG-3′, respectively) were from Microsynth AG. Fifty picomoles of each *siRNA* were transfected using TransfeX^TM^ (ATCC HB-8065TM, ATCC Manassas, VA, USA). For ectopic expression of C20orf204-189AA experiments, C20orf204-189AA cDNA was isolated from HepG2 RNA by RT-PCR using primers 5′-AAA GGA TCC ATG GTA CCC CCT AAG CCT GCA C-3′ and 5′-AAA AAG CTT GGA GTC CCT GGA GGC CGG GGC GCG-3′. The PCR-product was then cloned into pcDNA3.1 MycHis plasmid. Plasmids with or without *Linc00176* cDNA were transfected using TransfeX^TM^.

### Peptide-specific antibodies

Antibodies against the mixture of two synthetic peptides corresponding to 51–65 and 138–154 (amino acid numbers in C20orf204-189AA) were generated in rabbits by Kaneka Eurogentec S.A. (Seraing, Belgium). Two peptide columns were applied for further purification of 51–65 (numbers represent amino acid number, peptide I) and 148–164 (peptide II) specific antibodies.

### Cell viability testing with PI labeling followed by FACS analysis

HeLa cells were transfected with pcDNA3mycHis carrying C20orf204-189AA cDNA or empty vector and then transfectants were trypsinized and fixed in ice-cold ethanol. Fixed cells were stained in PI solution (100 µg/ml PI, 100 µg/ml RNAse, 0.05% Triton X-100) and analyzed by an Accuri-C6 flow cytometer (BD Biosciences, Heidelberg, Germany) under standard settings for detecting PI (FL-2) using a 488 nm laser.

### WST-1 assay

HeLa cells (500–2000 cells/well) were seeded in duplicate on a 96-well plate and then transfected with vector Crtl, C20orf204-189AA and C20orf204-(1-74AA) or C20orf204-(73-189AA) and incubated for 3 days. A WST-1 proliferation assay kit (Roche Diagnostics) was employed according to the manufacturer’s instructions.

### Crystal violet

HeLa cells (500–2000 cells/well) were seeded in duplicate on a 96-well plate and then transfected with vector Crtl, C20orf204-189AA, and C20orf204-(1-74AA) or C20orf204-(73-189AA) and incubated for 3 days. Cells were then washed with phosphate-buffered saline (PBS) and fixated with methanol. Crystal violet dye was applied for 10 min. After air drying the plate, the dye was solubilized in methanol and absorbance was measured at 595 nm.

### Immunohistochemistry/immunofluorescence

Immunohistochemical studies were performed as detailed previously^12^. Mouse monoclonal anti-Myc, GST, KDEL, and C23 antibodies were purchased from Santa Cruz Biotechnology (Santa Cruz, CA, USA). Rabbit monoclonal anti-Myc antibody was from Cell Signaling Technology (Cambridge, UK). Mouse monoclonal antifibrillarin antibody was from Abcam plc (Cambridge, UK). Confocal images were obtained using a Zeiss LSM700 confocal laser scanning microscope.

### In vitro transcription/translation

Radiolabeled substrates were generated by in vitro transcription/translation using the plasmids pSNAP-23his6 as a positive control and pcDNA3.1-C20orf204-189AA-MYC, the SP6/T7-coupled TNT reticulocyte lysate system (Promega), and [^35^S]methionine (370 kBq/μl, >37 TBq/mmol, Hartmann Analytic, Braunschweig, Germany) according to the manufacturer’s instructions.

### Immunoblotting procedures

Details of immunoblotting have been described previously^23^. Corresponding proteins were visualized by incubation with peroxidase-conjugated anti-mouse or anti-rabbit immunoglobulin, followed by incubation with SuperSignal West FemtoMaximum Sensitivity Substrate (Pierce, Rockford, IL, USA). Results were documented on a LAS4000 imaging system (GE Healthcare BioSciences, Uppsala, Sweden).

### Semi-quantitative RT-PCR and qRT-PCR analysis

RNA was isolated from cells with the High Pure RNA Isolation kit (Roche Diagnostics) or the ReliaPrep^TM^ miRNA cell and tissue miniprep system (Promega, Madison, WI, USA) according to the manufacturer’s instructions. one microgram of RNA was reverse-transcribed using oligo dT primer or random primer and the ProtoScript^®^II Reverse Transcriptase (New England Biolabs GmbH, Frankfurt am Main Germany) following the instructions provided. One-twentieth of the cDNA mix was used for real-time PCR with 10 pmol of forward and reverse primer and ORATM qPCR Green Rox kit (HighQu, Kraichtal, Germany) in a Qiagen Rotorgene machine. The levels of mRNA expression were standardized to the *glyceraldehyde-3 phosphate dehydrogenase (GAPDH)* or *beta-Actin (Actin)* mRNA level. Primer sequences are shown in Table S1.

### RNA immunoprecipitation

HepG2 cells were lysed with lysis buffer (10 mM Tris, 150 mM NaCl, 1 mM PMSF, 0.4% NP40, protease inhibitor cocktail (Sigma-Aldrich), and RNase inhibitor). After centrifugation, supernatants were incubated with control IgG or antinucleolin antibody, and were then precipitated with protein G Sepharose. Bound RNAs were isolated and treated with DNAse for 15 min, then analyzed by RT-PCR.

### GST pull-down assay

HepG2 cells were lysed with lysis buffer (10 mM Tris, 150 mM NaCl, 1 mM PMSF, 0.4% NP40, protease inhibitor cocktail (Sigma-Aldrich, Munich, Germany). After centrifugation, supernatants were incubated with GST, GST-C20orf204-189AA, GST-C20orf204-73-189AA, and GST-C20orf204-1-74AA fusion proteins. Bound proteins and bound RNAs were analyzed by nucleolin-specific immunoblot or rRNA-specific RT-PCR, respectively.

### Metabolic labeling and analysis of pre-rRNA transcription and processing

HeLa cells were incubated at 37 °C in 5 ml of DMEM without phosphate (Invitrogen) supplemented with 10% fetal calf serum. After 2 h, [32P] Pi (Hartmann Analytic) was added to the medium (125 µCi/ml) for the indicated times. At the end of the labeling, total RNA was extracted with the High Pure RNA isolation kit (Roche), separated by 1% agarose electrophoresis. Radioactivity was detected with a Fujifilm FLA-9000 scanner.

### Reciprocal pixel intensity

To quantitate the intensity of nucleolin staining, reciprocal pixel intensity was determined by subtracting nucleolin intensity (as measured by the mean intensity function in the Nikon NIS elements D 3.0 Software) in 315 and 348 cells from the maximum pixel intensity in white unstained areas of HeLa cell transfected control vector (Ctrl.) or vector carrying C20orf204 cDNA (C20orf204), respectively.

### Statistical analysis and limitation of the study

Cell experiments were performed in triplicate and a minimum of three independent experiments were evaluated. Data were reported as the mean value with standard deviation. The statistical significance of the difference between groups was determined by the Student’s test (two sided) or with the CHITEST Excel function in Table 1. Correlation between nucleolin expression level (log2 RSEM (RNA-Seq by Expectation-Maximization) ≥13.5 (*n* = 82): log2 RSEM < 13.5 (*n* = 289)) and survival time (within 7.5 years) was determined by log-rank test^24^.

Primary 366 HCC data gathering was limited by availability from the cancer genome atlas (TCGA) data (https://cancergenome.nih.gov/).

## Supplementary information

Supplemental Figures

## Data Availability

All data generated or analyzed during this study are included in this published article and its additional files.
