# Supplementary material for: C20orf204, a hepatocellular carcinoma-specific protein interacts with nucleolin and promotes cell proliferation
Source: Oncogenesis. 2021 Mar 17;10(3):31. doi: 10.1038/s41389-021-00320-3 (PMC7969625; doi:10.1038/s41389-021-00320-3)
Supplement: Supplementary file 1 — Supplemental Figures [file 41389_2021_320_MOESM1_ESM.pdf]

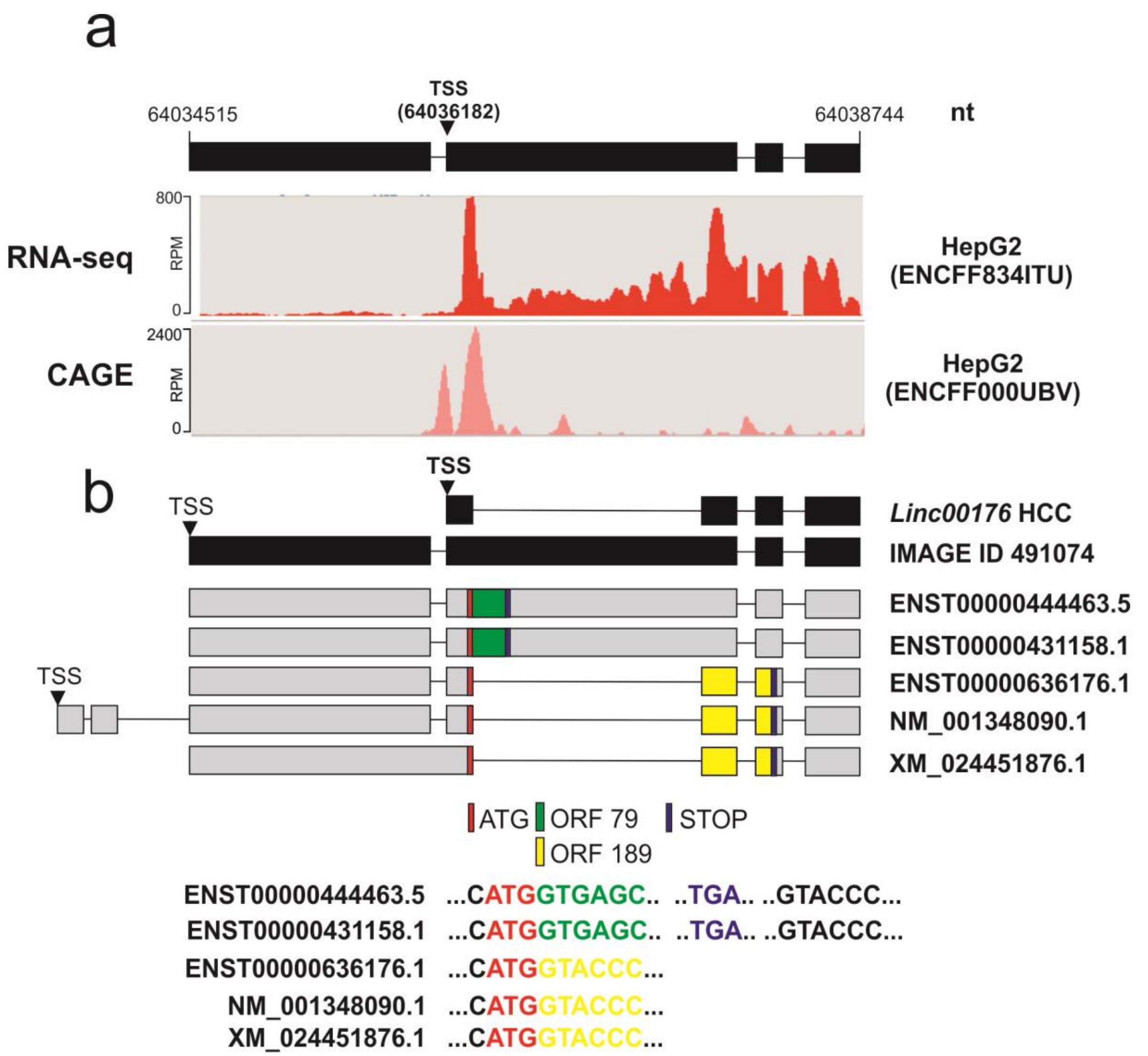

Fig. S1: a CAGE data shows a transcription start site (TSS) at nt 64036182 in HepG2 cell lines, corresponding to Linc00167 and no signal for other isoforms. b Sheme of Linc00176/C20orf204 (ENST00000444463.5, ENST00000431158.1, ENST00000636176.1, NM_001348090.1 and XM_024451876.1).

ENST00000636176.1 is identical to *Linc00176-HCC*, and ENST00000444463.5 is identical to IMAGE ID:4941074 encoding a peptide 189 amino acids long (yellow). Notably ENST00000444463.5 and ENST00000431158.1 encode a peptide only 79 amino acids long (green).

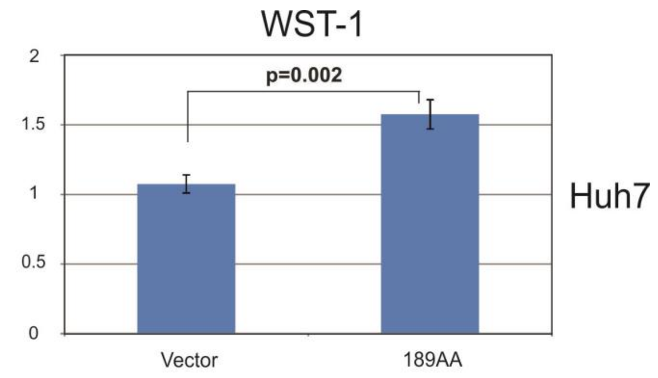

Fig S2. Huh7 cells were transfected with Vector or pcDNA3.1-C20orf204-189AA for 2 days and supplied for WST-1-assay.

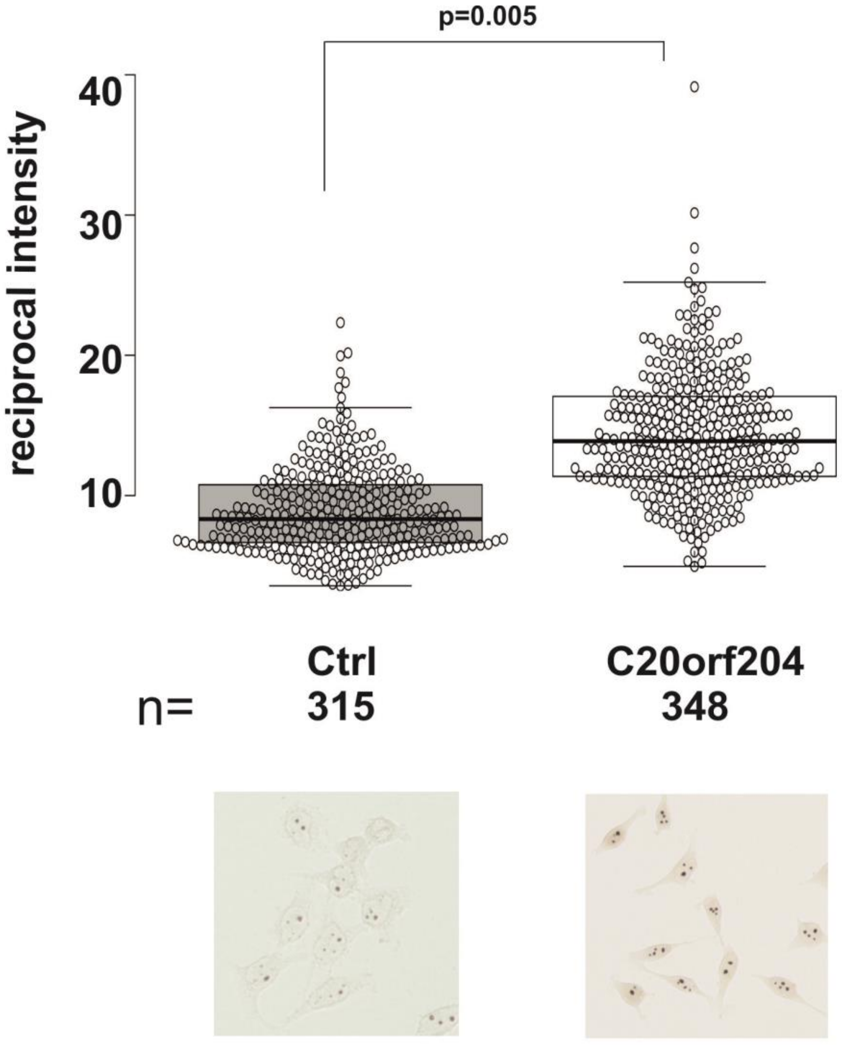

Fig. S3: HeLa cells were transfected, then fixed and IHC stained. To quantitate the intensity of nucleolin staining, reciprocal pixel intensity was determined by subtracting nucleolin intensity in 315 and 348 cells from the maximum pixel intensity inwhite unstained area from HeLa cells transfected with control vector (Ctrl) or vector carrying C20orf204 cDNA (C20orf204), respectively.

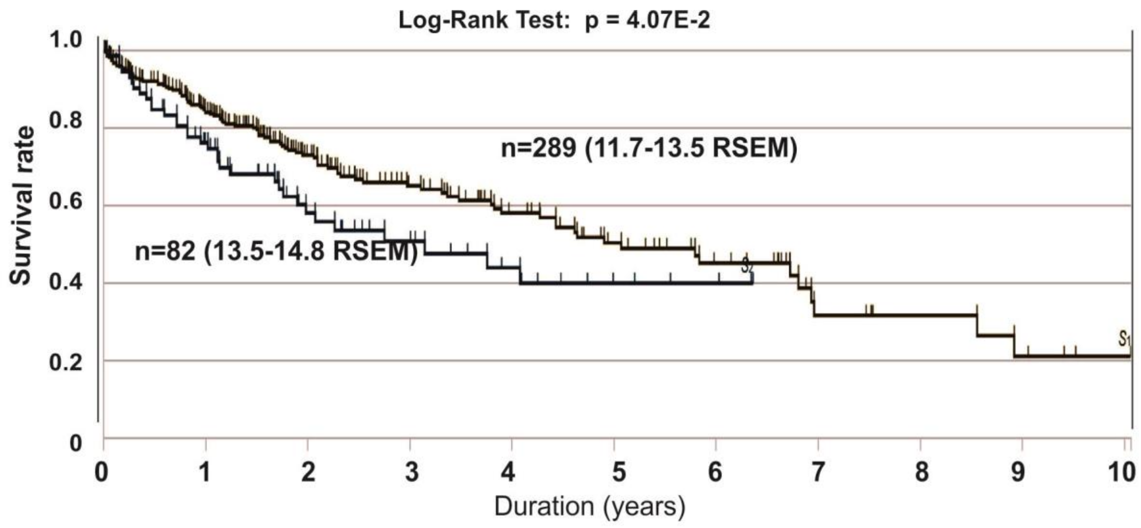

Fig. S4: Correlation between Nucleolin expression level (log2 RSEM (RNA-seq by expectation-maximization)≥ 13.5 (n=82): log2 RSEM <13.5 (n=289)) and survival time (with 10 years) is shown using Kaplan-Meier estimation (Log rank test: p=4.07e-2).

Table S1: PCR primer pair sequences for selected genes.

**Gene**	**Accession number**	**Forward primer**	**Reverse primer**
Gapdh	NM_002046	TGTTGCCATCAATGACCCCTT	CTCCACGACGTACTCAGCG
C20orf204 F1Linc00176-E1 (F1-R1)	NR_027686.1	ACTCCTGTCACCTGTGTGGT	GGCTGAAGAGGTAAGTCTGAGG
C20orf204 4Ea (F2-R2)	NR_027686.1	CAGGGACTCCTAGCGCGG	AGCGCCAGCTCCGTTTATTG
C20orf204 4Eb (F3-R3)	NR_027686.1	ATGTATTTAGTCTGTGCAACGG	CAAGGCTCTGAAGTCCAGGA
Nucleolin	NM_005381.3	GGTGGTCGTTTCCCCAACAAA	GCCAGGTGTGGTAACTGCT
rRNA 28S	NR_046235.3	GGGATTATGACTGAACGCC	AACGTGCGGTGCGTGAC

Gapdh: glyceraldehyde-3 phosphate dehydrogenase (202 nt); C20orf204: (243 nt); -E1 (296 nt); C20orf204-E4a (243 nt); C20orf204-E4b (199 nt); Nucleolin (135 nt); rRNA 28s (269 nt)
